# Supplementary material for: Are carbon emissions trading and green financial instruments synergistic? -Comprehensive quantitative research based on content analysis
Source: PLoS One. 2024 Mar 7;19(3):e0298601. doi: 10.1371/journal.pone.0298601 (PMC10919601; doi:10.1371/journal.pone.0298601)
Supplement: S1 Data — (ZIP) [file pone.0298601.s001.zip › Data/地级市Z_F工作报告.zip!/╣≤╓▌/╣≤╤⌠╩╨/└┤╫╘╣1⁄2╓┌║┼í╛┬φ┐╦╩2╛▌═°í┐_2008.docx]

各位代表：

现在，我代表市人民政府向大会作工作报告，请予审议，并请各位政协委员和其他列席人员提出意见。

一、2007年工作回顾

2007年，我们高举中国特色社会主义伟大旗帜，坚持以邓小平理论和“三个代表”重要思想为指导，深入贯彻落实科学发展观，在省委、省政府和市委的正确领导下，在各级人大、政协的监督和帮助下，团结和依靠全市人民，开拓创新、锐意进取，全市经济、社会、政治、文化、生态和城乡建设朝着更加协调的方向迈进，圆满完成了市十二届人大一次会议确定的目标任务。

（一）着力转变发展方式，国民经济又好又快发展。全市生产总值达696.4亿元，同比增长15.8%，是1984年以来的最高增速。其中，一产完成增加值45.07亿元，同比增长8.2%；二产完成增加值325.15亿元，同比增长16.3%；三产完成增加值326.18亿元，同比增长16.2%。财政总收入完成188.66亿元，同比增长19.7%；地方财政收入完成75.92亿元，同比增长23.3%。全市固定资产投资完成500.38亿元，同比增长21.1%，纳入跟踪管理的51个重点项目建设达到预期目标。能耗水平继续下降，列入考核的规模以上企业万元工业总产值综合能耗为1.73吨标准煤，比全年目标值2.22吨标准煤低22.07%。工业固体废弃物综合利用190万吨，二氧化硫和化学需氧量排放量均比上年有所下降，完成了年初确定的目标任务。

（二）着力改善民生，人民群众更多地共享发展成果。坚持优先发展教育，对农村义务教育阶段学生全面实行“两免一补”，使受惠学生从上年的46.9万人增至57.6万人。对8万名进城务工人员子女实行义务教育补贴，完成32所农村寄宿制学校建设，启动9所农村标准化学校建设，改扩建接收农民工子女入学的公办学校8所。统筹城乡卫生事业发展，全面实施新一轮农村初级卫生保健，继续推行新型农村合作医疗制度，农民参合率从上年的72.6%提高到92.63％；建成三所惠民医院，完成10个标准示范性社区卫生服务中心、8个乡镇卫生院改造和187个村卫生室建设。村村通广播电视工程解决了39925户农民看电视、听广播难的问题，全市20户以上自然村通广播电视率达到100%。劳动就业规模继续扩大，社会保障体系不断完善。实现新增城镇就业岗位49919个，城乡统筹就业74867人，城镇登记失业率为3.76％。切实抓好“零就业家庭”就业援助工作，实现动态消除“零就业家庭”。开展城镇居民基本医疗保险试点工作，25.17万居民参保登记。离退休养老保险、下岗职工基本生活费实现100％按时足额发放，城乡低保实现应保尽保，低收入群体和特困家庭基本生活得到保障。启动“住有所居”行动计划，1000户住房困难群众得到了住房保障和安排了廉租房，开工建设5万平方米廉租房。加强食品药品质量监管，在全国省会城市食品放心工程综合评价中获得优秀等次。扎实推进“平安贵阳”工程，加强社会治安防控体系建设，集中开展“严打两抢一盗，保卫百姓平安”专项行动，社会治安状况进一步好转，市民安全感明显增强。进一步完善突发公共事件应急体系，全面落实安全生产责任制，实现了事故和死亡人数“双下降”。扶贫攻坚取得新成效，完成易地搬迁扶贫250户共1200人。农村富余劳动力培训转移有序进行，转移农村富余劳动力2.6万人。城乡居民收入水平明显提高，城市居民人均可支配收入达12781元，同比增长13.9%；农民人均纯收入达4088元，同比增长18.8%。为民办好的十件实事全面完成。

（三）着力优化产业结构，经济运行质量显著提高。坚持消费带动，着力发展旅游业和现代服务业，第三产业在经济发展中的比重日益上升。实现社会消费品零售总额279.39亿元，同比增长18.9%。居民消费价格指数上涨5.1%，涨幅在全省处于较低水平。建成18个农贸市场、2个早菜批发市场和450个农家店。在全省率先实现生猪机械化集中屠宰。成功举办“森林之城·魅力贵阳”2007中国首届避暑节系列活动，旅游业呈现良好发展势头。接待国内游客2314.14万人次，同比增长25.4%；海外游客12.09万人次，同比增长35.6%；旅游创汇5872.28万美元，同比增长50.4%；实现旅游总收入125.28亿元，同比增长48.2%。大力实施“工业强市”战略，工业经济持续较快增长。完成规模以上工业增加值244.66亿元，同比增长15.1%；规模以上工业产销率达96.4%，实现利润总额58.02亿元，同比增长24.5%。产业集中度和关联度进一步提高，优势产品产量大幅增长，重点行业产值快速提升。“险峰”牌数控磨床等3个产品获得中国名牌产品称号。同济堂药业成功在美国上市。新农村建设扎实推进，制定并实施《关于统筹城乡发展加快推进城乡一体化建设的决定》及相关配套政策措施，城乡一体化起步良好。水库除险加固、万户小水池、四改一气、农村危房改造、村寨串户路建设等项目全面完工，解决了15万农村群众的饮水安全。蔬菜、花卉、果树、中药材、奶牛、生猪、蛋鸡等产业获得较大发展。引进广东温氏、江苏鼎球绢丝纺等国内知名龙头企业，命名市级以上龙头企业75个，扶持农业专业合作经济组织371个。

（四）着力巩固生态优势，城乡生态文明意识增强。扎实抓好以营林工程和绿化建设为重点的生态建设，出台城市“绿线”管理办法，开展严厉打击违法占用林地专项行动，拆除违法占用林地建房35357平方米，新增城市绿地32.7万平方米，全市森林覆盖率达39.19％。循环经济建设稳步推进，完成市级试点项目12个。初步建成再生资源回收利用体系。节能减排工作力度进一步加大。提前关停华电清镇发电公司2×6.5万千瓦机组，对年耗能3000吨标准煤以上的113户企业加强了监管，淘汰落后产能企业88户。实施水晶集团、贵阳煤气气源厂等企业的14个重点节能减排技改项目，确定10户企业为第三批清洁生产试点企业。加快重点污染源的在线监测，在中心城区开展了以治理噪声、饮食油烟污染为主的“双降”工作。全年大气质量良好以上天数达346天，比上年增加7天。全面启动“依法治理两湖一库，确保市民饮水安全”工作，初步建立了“两湖一库”治理保护的长效机制。

（五）着力完善城市功能，城市建设和管理取得新成绩。大力改善城市基础设施条件，纳入计划实施的25个市政基础设施项目进展顺利。环城高速公路南环线从前期准备到开工创下贵州高速公路申报审批时间的最快纪录，贵遵高速公路扎南段建成通车。开工建设花溪二道、北京西路、油小线、贵金线、新庄污水处理厂、小河污水处理厂（二期）等事关全局的基础设施项目。1000公里农村公路建设、服务大楼立交改造、3个交通环岛环境综合整治工程及10个人行过街系统建成并投入使用。环城高速公路西南环线等重点项目按计划实施。朝阳洞路、轻轨交通等项目前期工作进展顺利。金阳新区建设步伐加快。省广播电视大学金阳新校区、省气象综合信息遥感楼项目开工建设，金阳医院建成投入使用，金朱西路、奥体中心和“文化山”等项目有序推进。完成101个历年滚存财政性投资项目“投转固”工作，支付使用资产价值59.2亿元，切实加强城市规划和管理。成立市城乡规划建设委员会，新一轮城市总体规划修编工作加快进行，完成三桥马王庙片区行政区划调整和控制性详规、“一环两路”景观综合整治规划等。“整脏治乱”工作深入推进，“城中村”整治工作取得成效。顺利完成城区四条街道临时占道百货夜市摊点的清退工作，户外广告牌匾、环境卫生、违法建筑整治工作效果明显，市容市貌进一步改善。启动实施城区“畅通工程”，有效缓解了城区行路难、行车难、停车难等状况。

（六）着力推动改革开放，经济发展活力彰显。国企改革顺利推进，完成50户企业改革重组工作。着力清除体制机制障碍，推动非公有制经济快速健康发展。稳步推进农村综合配套改革试点，“乡财县管乡用”、“村财乡代管村用”试点改革工作进展顺利。农村公路管理养护改革不断深化，集体林权制度改革试点工作基本结束。深入推进国库集中收付制度改革、预算指标管理系统改革。成功举办赴新加坡和马来西亚旅游投资促进活动、赴上海和苏州招商推介活动及第三届中医药博览会，组织参加贵州省赴广州和深圳经贸洽谈等大型招商活动。加强与成渝经济区、泛珠三角经济圈内经济体的合作，积极融入东盟自由贸易区，贵阳城市经济圈合作进一步加强。经济发展外向度进一步拓展，引进投资额5000万元以上大项目达101个。直接利用外资达8100万美元，同比增长15.5％；内资到位资金达252亿元，同比增长20.2％。进出口总额达16.92亿美元，同比增长51.3％，其中出口增长53.8%。

（七）着力发展社会事业，经济社会协调发展。科技创新能力进一步提高。连续第四次荣获国家科技进步考核先进城市称号，进入国家知识产权示范城市创建市行列，以实施重大科技项目为支撑，共实施市级科技项目139个，完成16项重大科技攻关项目，新增6家省级技术中心和18家企业工程技术中心，新建贵阳国际生物材料产业化公共实验室、生命科学基础实验平台和太阳能级多晶硅中试基地，合作建立贵州大学贵阳研究院。职业教育发展势头良好。启动市职教中心建设，贵阳职业技术学院和贵阳护理职业学院正式挂牌，“一本两专”的高等教育格局初步形成。高质量通过国家一类城市语言文字工作评估。文化体育事业蓬勃发展。成功举办第六届中国舞蹈荷花奖民族民间舞比赛，选送的《水姑娘》等节目分获金、银、铜奖等，实现在国家级舞蹈大赛中“零的突破”。《布依女人》获“国家舞台艺术精品工程”提名奖。广播电视数字化改造成效显著。成功举办首届亚洲青年动漫大赛，建成贵阳数字内容产业园。组队参加了全国第六届城市运动会。第五次荣获“全国双拥模范城”称号。继续保持稳定的低生育水平，人口出生率为10.33‰，人口自然增长率为4.83‰，符合政策生育率为97％。司法、统计、审计、工商、物价、人防、新闻出版、社会科学、外事侨务、民族、宗教、老龄、残联、对台、市志、档案等各项事业取得明显进步。

（八）着力推进依法行政，政府自身建设呈现新气象。认真执行人大及其常委会的决议、决定，自觉接受人大及其常委会的法律监督、工作监督和政协的民主监督。办理人大代表建议196件，政协提案461件，办复率均达100%，办理满意率分别达97%和99%。实行政府重大事项听证会制度，对环城高速公路建设、畅通工程、两湖一库治理保护等事关发展大局、与老百姓生活密切相关的工作，主动听取、充分吸纳人大、政协和社会各界的意见和建议，努力提高政府决策的科学化、民主化水平。全面推进依法治市工作，政府依法行政意识明显增强。强化廉政建设，全面实行领导干部任期经济责任审计制度，机关作风教育整顿、治理商业贿赂等工作取得新进展，大力惩处了一批腐败分子。进一步规范行政审批程序，落实各项便民利民措施，整合成立贵阳市政务服务中心并正式运行。

各位代表，2007年是本届政府任期第一年。在这一年里，“中国避暑之都”、“中国舞蹈之都”城市品牌落户贵阳，全市综合经济实力、投资环境再上新台阶，贵阳市在推动历史性跨越的进程中迈出了坚实的一步。这些成绩的取得，是省委、省政府、市委正确领导和市人大、市政协有效监督的结果；是各级各部门解放思想，开拓创新，提高效能的结果；是全市人民团结一致，顽强拼搏，真抓实干的结果。在此，我代表市人民政府，向全市广大工人、农民、知识分子、干部群众致以崇高的敬意!向各民主党派、工商联、无党派人士和人民团体，向驻筑人民解放军指战员、武警部队官兵、公安政法干警，向所有关心支持贵阳建设和发展的各界朋友，表示衷心的感谢!

在充分肯定成绩的同时，我们也清醒地看到，在经济社会发展中仍存在一些矛盾和问题：一是经济发展方式还较为粗放，支撑大发展的规模工业体系尚未形成，产业结构仍不合理，经济持续较快发展的基础还不够稳固。二是城镇化水平总体偏低，城市配套服务功能不够完善，城市对促进经济发展的承载力和辐射力还有待增强。三是农业基础条件较为薄弱，农业龙头企业带动能力不强，农业产业化水平不高，农民增收致富的渠道比较单一，持续增收的难度较大。四是经济发展与资源、环境之间的矛盾仍然突出，土地资源利用、节能减排的压力将不断增大。五是公共服务和社会管理中还存在薄弱环节，就业、社保、上学、就医、社会治安、自然灾害防御、公共卫生和公共安全处置等方面存在的问题有待加速解决。六是财政进入偿债高峰期，财政支出压力增大，财政调控能力相应减弱。七是受食品类价格上涨的影响，居民消费价格指数持续攀升，对城市低收入群体和农村贫困人口生活带来较大影响。对这些问题，我们一定在今后的工作中采取切实可行的措施，努力加以解决。

二、今年经济社会发展的奋斗目标和任务

2008年经济社会发展的总体要求是：高举中国特色社会主义伟大旗帜，以邓小平理论和“三个代表”重要思想为指导，深入贯彻落实科学发展观，全面贯彻落实党的十七大、省委十届二次全会和市委八届四次全会精神，以建设生态文明城市为总目标，坚持好字优先、能快则快，进一步解放思想，推动科学发展，促进社会和谐，加快转变经济发展方式，推动经济结构优化升级，全面推进改革开放，切实加强生态环境保护，更加重视节能减排和自主创新，更加重视社会建设和改善民生，努力提高发展的质量和效益，促进经济又好又快发展，着力增强综合竞争力，为建设生态文明城市开好局、起好步。

2008年经济社会发展的原则是：1、坚持好字优先、能快则快，努力推动经济又好又快发展。我们将按照胡锦涛总书记“对于像贵州这样的西部省区，只要符合科学发展观的要求，有条件、有效益，就要努力加快发展”的指示，始终将“做表率、走前列”作为各级政府的政治责任和工作追求，将抢抓机遇、加快发展作为全部工作的根本主题，将好字当头、好中求快，以好带快、以快促好作为经济工作的根本原则，切实把经济发展转入科学发展的轨道。2、树立保住青山绿水也是政绩的发展理念，进一步巩固和增强生态优势。我们将正确处理开发与保护的关系，把建设资源节约型、环境友好型社会放在工业化、现代化发展战略的突出位置，培育城市精神，加强城乡基础设施建设，发展生态产业，逐渐形成节约资源、能源和保护生态环境的产业结构、消费模式、发展方式。3、大力优化经济结构，着力转变经济发展方式。建设生态文明城市必须大力转变经济发展方式，目的是实现更好更快的发展。我们将加快发展生态产业，努力促进服务业优化升级和提高比重，扎实抓好自主创新和节能减排工作，推进生态文化建设，实现经济发展方式的真转真变。4、着力改善民生、服务群众，切实实现好、维护好、发展好最广大人民的根本利益。我们将坚持“民生是最大的公务”，以“人民群众生活中的小事，就是政府工作的大事；人民群众生活中的难点，就是政府工作的重点”作为工作要求，大力实施“六有”民生行动计划，努力适应和满足人民群众的新要求、新期待，把解决人民群众最关心、最直接、最现实的利益问题与建立长效机制统一起来，量力而行，尽力而为，更加注重改善困难群众和低收入群众生活，推动公共资源配置更多向群众直接受益的方面倾斜，使发展成果惠及全市人民。

2008年经济社会发展的主要预期目标是：全市生产总值增长13%以上，其中，第一产业增加值增长7%；第二产业增加值增长14%；第三产业增加值增长15%。财政总收入增长13%，地方财政收入增长14%。全社会固定资产投资增长18%。外贸进出口总额增长20%。社会消费品零售总额增长15%。城市居民人均可支配收入增长8%，农民人均纯收入增长10%。城镇登记失业率控制在4%以内。单位生产总值综合能耗降低4.5%，主要污染物排放减少2%。

2008年为民办好的十件实事是：（一）确保进城务工人员子女接受义务教育；新增城镇就业岗位4万个；完成农村劳动力非农技能培训1.8万人次，转移就业1.44万人；力争开工建设贵阳职业技术学院；建成贵阳护理职业学院金阳新校区并实现搬迁。（二）全面推行城乡医疗救助，惠民医疗政策普及到县级人民医院、乡（镇）卫生院、社区卫生服务中心（站）；在全市范围内实施城镇居民基本医疗保险；开工建设市妇幼保健院综合病房大楼；改造10个乡（镇）卫生院。（三）启动新型农村社会养老保险制度；继续推进贵阳市社会化养老工程；建成市流浪未成年人救助保护中心主体工程。（四）竣工经济适用住房100万平方米；改造农村危房6928户；开工建设廉租住房10万平方米，建成5万平方米，使符合条件的城市低保家庭享受廉租住房保障政策达到应保尽保。（五）继续开展“严打两抢一盗，保卫百姓平安”专项行动，完成10万个城市中心区110群众报警定位标志安装和三江、桐木岭2个戒毒康复农场主体工程；启动烂泥沟看守所整体搬迁工作。（六）建设或改造8条城市道路及3处人行天桥；在9个人行地下通道新建残疾人无障碍设施；新增3000个停车位；开通3条夜间公交线路；背街小巷亮灯率达98%。（七）着力改善“两湖一库”水质环境；南明河、黔灵湖水质进一步好转；全市森林覆盖率提高一个百分点；云岩区、南明区各建成1个中心绿化广场；建成环境保护法庭办公楼。（八）解决农村20万人饮水安全；建成全市“四改一气”共17600套以及后续服务网点200个、村寨串户路1000公里；新增电视入户2.1万户，建成自然村广播站500个，使农村电视入户率达88%以上；建立无公害蔬菜基地100个；新建标准化奶牛养殖小区10个；改造建设农家店100个；新建村级综合楼127个；在云岩区、南明区各建1个标准绿色农贸市场。（九）森林公园、南郊公园免费开放；取消全市所有公厕收费。（十）在金阳开工建设贵阳奥林匹克中心主体育场等社会基础设施项目；启动汉湘街改造；启动贵阳市市民生态型公益性公墓建设。

围绕上述要求、原则、目标和实事，我们将重点抓好八项工作：

（一）扎实推进产业结构优化升级，构筑生态产业体系

加快服务业发展。完善服务业发展的政策措施，不断提升第三产业比重和发展水平。一是积极扩大消费。拓宽城乡居民收入渠道，按照有关规定规范发放公务员津补贴，抓好最低工资标准的实施，增强居民消费能力。大力培育旅游、住房、汽车、文化、体育、休闲娱乐等消费热点，推动消费结构升级。建立公开、平等、规范的行业监管制度，规范服务市场秩序，切实维护消费者合法权益。二是大力发展旅游业。以举办“贵阳避暑季”为载体，全力推介贵阳“中国避暑之都”城市名片，发展温泉旅游，努力将我市打造成世界著名的避暑旅游城市和“温泉之城”。加大旅游市场开拓和营销力度，提升青岩古镇、天河潭景区、开阳“十里画廊”、南江大峡谷、阳明文化遗址、乌江峡红色景区、修文野生动物园等旅游景区景点品质，积极发展乡村特色旅游，加大地方特色旅游产品培育开发力度，加快完善交通、酒店、餐饮、购物、通信等旅游配套设施，全面推进景区景点所有权、经营权、管理权分离，把旅游产业培育成全市经济发展的重要支柱产业。三是加快发展现代服务业。突出发展金融、保险、咨询、创意、医疗保健服务等新兴服务业。积极发展现代物流业，抓紧实施《贵阳市现代物流业发展规划》，加快引进国内外大型物流企业，重点发展矿产加工、医药、食品、高新技术等4类特色行业物流，抓好二戈寨、金阳、扎佐等物流园区的规划和建设，尽快将贵阳建设成为大西南物流枢纽和分拨中心。四是加强和完善市场体系建设。实施大市场工程，重点抓好谷丰粮油批发市场、三桥综合批发市场、浙江商城、西南家居装饰城等10个市场的升级改造，稳步推进大型购物中心、连锁超市的建设。抓好合群路、箭道街美食一条街和其他风味小吃街的规划和建设，培育“黔菜”品牌，支持发展地方特色名小吃连锁经营。完成25家农贸市场的改造升级，争创1—2个国家级商业示范社区，推动零售业加快发展。加快农村市场体系建设，继续推进“万村千乡”和“双百”市场工程。培育发展便利型超市、家政、养老等服务行业，形成较为完善的社区服务网络。

大力推进新型工业化。把提高自主创新能力作为工业结构调整的中心环节，加快发展带动力强、产业链长、高增长性的产业。一是加快发展支柱产业和特色产业。以神奇、益佰、同济堂、健兴等企业为龙头，推进贵阳现代中药企业整合。大力支持老干妈公司等特色食品企业进一步发展壮大，不断提升现代中药和特色食品产业整体实力。打造汽车、工程机械、特种数控机床整机及零部件、航空航天零部件、石油专用机械及成套设备等产业链，提升先进制造与新型装备制造业发展水平。积极推进贵阳卷烟厂异地搬迁、华电清镇发电公司2×60万千瓦机组、西南工具厂高精度数控铣刀生产线等重点续建项目建设，开工建设贵阳海信电子有限公司平板电视产业化二期、贵航集团小河军民高新技术产业园等重大产业化项目，完成永红航空机械公司散热器生产线、三占集团公司高品质钎钢具生产线等项目建设并投产达产。大力发展磷深加工、铝深加工和煤化工产业，完成开阳磷煤化工基地开磷集团120万吨/年磷酸二胺项目一期60万吨/年工程建设，推进白云铝深加工基地和一批煤化工项目建设。二是大力发展高新技术产业。在电子信息产品、新材料、光机电一体化等重点领域发展优势产业。加快新能源、节能环保等领域的开发和利用。在金阳科技园、新天工业园、数字内容产业园、汽车零部件工业园等重点区域形成若干新的高新技术产业集群，高技术产业产值比上年增长20%，软件产业产值增长50%。力争形成1个100亿元级特大型企业、3个50亿元级中型企业和一批10亿元级“小巨人”企业。三是加快园区建设。进一步理顺和完善高新开发区管理体制，规划建设麦架——沙文——扎佐高新技术生态产业经济带，实现高新开发区的扩容增效。加快小河——孟关装备制造业产业带基础设施建设。四是加大技术改造力度。重点抓好险峰机床厂、大力士轮胎公司高性能载重子午线轮胎生产线二期等技术改造项目，加大铝、磷、煤等资源型产业的重点能耗企业节能改造力度，推动工业结构优化升级和能耗水平进一步下降。

以生态功能为基础优化城市生产力布局。编制完成《贵阳市生态功能区划》，明确各生态区和各区（市、县）的功能定位。中心城区重点发展服务业、高新技术产业和先进制造业。近郊区实行重点突破、分片发展，新天片区重点发展高新技术、温泉旅游产业，龙洞堡片区重点发展物流业和临空经济，花溪片区重点发展旅游、文化产业。加快推进清镇、修文、息烽、开阳撤县（市）设区工作，重点发展旅游业、现代农业和加工业。整合贵阳城市经济圈内基础设施、市场、产业优势，强力推进经济强县建设，巩固和提升我市在全省的领先地位。

(二)扎实推进城市基础建设和管理，精心打造宜居宜业宜游城市

坚持规划引领。充分发挥市城乡规划建设委员会的作用，严格按法定程序编制、审查和批准规划，强化对规划实施过程的监督管理，严肃查处违法建设行为。以建设生态文明城市为目标，以超前的意识做好新一轮城市总体规划的修编，完成龙洞堡临空经济区、环城高速公路沿线、贵广快速铁路新客站等的规划工作，加快贵阳城市化进程，使城市化率比上年提高一个百分点。

提升城市品质。继续抓好贵黄路、贵遵路、东出口高速公路、花溪大道沿线环境综合整治，启动并完成沙冲路街景整治。进一步加大社区绿化、山体公园建设的力度，在城区主干道沿线实行立体绿化、美化，彰显林城特色和魅力。金阳新区公共设施建设加快向40平方公里规划范围拓展，促进金阳与三桥马王庙片区、白云的联动发展。全力配合完成清金线、清纳线征地拆迁工作，抓好金朱西路、长岭北路等道路建设，加快奥林匹克中心主体育场、会展中心、交警指挥中心、科技馆、档案馆等一批项目建设。观山公园正式向公众开放。加快金阳中央商务区、商贸走廊的建设步伐，强力推动金阳新区产业发展和人气、商气聚集。

实施“畅通工程”。积极配合做好厦蓉高速公路贵阳段、贵广快速铁路新客站、贵阳铁路枢纽等工程建设。力争启动城市轻轨试验段建设。积极推进环城高速路西南环线及南环线、水东路、北京西路、花溪二道、油小线、贵金线、沙冲路及延伸段、开发大道延伸段等重大项目建设。加快黔江路、珠江路、朝阳洞路、南横街、贵医街、六广门体育场路、新华印刷厂支路等城市次干道和支路建设。努力完善城市交通基础设施体系。加快人行过街系统、智能交通监控系统、无障碍设施、停车场（位）建设，优先发展城市公交，解决百姓“出行难”、“乘车难”问题。

强化精细管理。进一步加大整脏治乱工作力度，对城区“五脏五乱”整治实行长效管理和网格化管理。实施中华路改造和重要路段、节点的夜景照明工程，有条件的背街小巷全部安装路灯。加大城市综合执法力度，强化对环境卫生、噪声管制、垃圾无害化处理和户外广告等的规范化管理，持续开展严厉打击制假售假特别是制售假证专项行动，大力整治城市“牛皮癣”等影响市容市貌的城市顽疾，塑造优美的城市形象。认真落实城市“绿线”管理办法，坚决制止开山毁林修建违法建筑行为，做好交通干道沿线可视范围内损毁山体修复工作。继续加强“城中村”改造，加大违法建筑的查处力度，完成“拆违”50万平方米。规范房地产市场，严厉打击违法圈地、占地行为。继续加大建筑市场、燃气市场的监管力度。全力配合做好缅气入黔工作，加强煤气气源点的建设。

（三）扎实推进城乡一体化，加快社会主义新农村建设

大力发展现代生态农业。认真贯彻落实2008年中央1号文件精神，实行公共基础设施向农村延伸，公共财政向农村覆盖，公共服务向农村倾斜，扩大农民就业空间，切实繁荣农村、服务农业、富裕农民，加快农业产业化步伐，促进城乡一体化发展。以城郊特色农业、都市特色农业为重点，加快发展猪——沼——蔬（果）等链式生态农业，建设一批生态农业示范园和绿色生态基地。大力实施畜、禽、蛋、奶、花、药、茶、果、蔬、桑等十大现代农业示范工程，新建养殖小区100个。建设中药材基地2万亩，茶园2.5万亩，果树基地1.8万亩，桑1.8万亩，无公害蔬菜基地50万亩，新建蔬菜批发市场1个，实现外销蔬菜60万吨。新增花卉大棚2100个，实现年产量2.5亿枝。大力扶持、培育、发展龙头企业，新增20个市级龙头企业，引进3—5家知名龙头企业。突出重点抓好专业批发市场及乡村旅游示范点建设，新增一批物流配送中心、农村超市和农家店，搞活农村市场流通。

努力提高农民自我发展能力。做好非农技能培训和农村富余劳动力有序转移就业，提高农民的创业和致富能力。认真抓好整村推进、产业化扶贫和贫困地区劳动力转移工作，投入贴息资金帮助贫困农户发展生产，切实改善贫困地区的基本生产生活条件，拓宽基本增收门路，提高贫困人口的基本素质。

夯实城乡基础发展条件。加大县乡公路、贫困村道路和村寨串户路改造力度，完成500公里农村公路改造，开工建设乡镇客车站20个。基本完成农村沼气池建设，完成中低产田土改造4.26万亩，基本农田建设2万亩。开工建设亚行贷款水利工程和7座小型水库，实施40平方公里水土流失治理。积极推进农村信息化建设。

加大小城镇建设和村庄整治工作力度。加快县城和重点镇的规划和建设，继续抓好43个新农村建设试点工作，重点抓好以白云麦架镇、乌当东风镇、“两湖一库”周边、乌当羊昌镇至开阳禾丰乡沿线的村庄整治工作，打造15个各具特色的自然村寨。积极抓好修文、息烽、开阳污水处理厂建设，完善城镇体系和配套功能。进一步理顺城乡一体化的体制机制。以改革小城镇户籍管理制度为突破口，逐步建立城乡统一的户口登记管理制度，加快建立城乡一体的人力资源市场和农村富余劳动力培训、转移就业机制，梯度引导农民向城镇集中。

（四）扎实推进生态环境建设和保护，着力构建资源节约型和环境友好型社会

大力发展循环经济。加快修订《贵阳市发展循环经济条例》，完善发展循环经济的体制和机制，推动产业循环式组合、企业循环式生产、资源循环式利用。加快开阳、息烽、清镇、白云等循环经济生态工业示范基地建设和修文扎佐医药园区生态化转型。继续实施循环经济项目试点和示范工程，重点抓好黄磷尾气生产甲酸钠、甲酰胺和1.5万吨超细粉体等工业项目，养殖业大型沼气、秸杆燃料、有机农肥等农业类项目。抓好再生资源回收利用体系项目、城市生活垃圾分装和资源化利用、金阳新区建筑行业循环经济试点等社会效益好的项目，使循环经济在加快生态文明城市建设中发挥更大的作用。

加大节能减排和清洁生产工作力度。全面落实《贵阳市节能减排综合工作方案》，严格实施污染物排放总量控制，重点抓好年耗3000吨标准煤以上工业企业的节能降耗工作，继续推进第三批试点企业实施清洁生产，强化清洁生产审核，加大对节能新技术、新产品、新工艺的推广应用力度，禁止新上高能耗、高污染的项目，坚决关停一批高能耗、高污染的企业，积极开展工业“三废”、农业废弃物、森林“三剩物”、建筑材料等废弃物的综合利用，确保完成年度节能减排计划。健全和完善政府推动、企业实施、全社会共同参与的节能减排工作机制，强化执法监察和监测，开展“节能减排全民行动”，为节能减排和清洁生产工作营造良好的社会氛围。

加强生态建设和资源保护。积极探索建立生态环境保护补偿机制，支持各种生态环保项目、替代产业和替代能源发展项目及生态移民项目建设。大力开展天然林保护、退耕还林、石漠化治理等工程，完成植树造林6.27万亩。启动环湖林带建设。加强森林资源的保护，严厉打击违法占用林地的行为。继续实施以治理“两湖一库”为重点的治水工程，加强花溪水库、松柏山水库等饮用水源的环境治理和保护，加大对花溪十里河滩的保护力度，加快南明河水口寺到新庄段截污沟、新庄污水处理厂、小河污水处理厂（二期）及二桥污水处理厂（二期）建设。严格实施污染物排放总量控制，健全完善排污许可和环境影响评价制度。加快建设全省危险废物暨医疗废物处理处置中心等项目。组织开展土地利用总体规划修编和第二次土地利用调查工作，实行最严格的耕地保护。严厉打击和查处无证勘查、开采和越界开采矿产资源等违法行为，加大地质灾害防治力度，促进矿山生态恢复。

（五）扎实推进改革开放，激活全社会创业动力

加快重点领域各项改革，不断消除影响发展的体制机制障碍。一是深化国有企业改革。确保完成政策性破产和50户以上国有企业改革重组工作，全力支持险峰实业总公司、公交总公司、供水总公司、商业储运（集团）有限公司等企业实行股份制改造，积极推进华能焦化制气有限公司等一批企业上市。在整合国有资产资源的基础上，组建一批新的投融资平台。推行国有及国有控股企业的财务经营预算制度，实现国有资产保值增值。二是加快财政体制改革。以强化财政预算刚性为重点，进一步深化部门预算管理、政府采购和非税收入管理等改革，完善公共财政体系。三是抓好项目管理改革。大力推行非经营性政府投资项目“代建制”，严格控制项目投资质量、成本和工期，建立和完善政府投资项目的科学决策机制、风险管理机制、防范机制和偿债机制。继续抓好“投转固”工作，形成“建成一项，转固一项”的良性接续机制。四是推进农村综合改革。重点做好“乡财县管乡用”改革试点工作，切实抓好乡镇机构改革、农村义务教育体制改革和新型农村合作医疗制度改革，全面推进集体林权制度改革，加快推进农村信用社、粮食流通体制、供销社、水利产权制度等方面的改革。

创新投融资体制机制，促进投资稳定增长。按照有保有压、突出重点的要求，继续抢抓国家实施西部大开发的机遇，加大固定资产特别是以交通和水利为重点的基础设施建设的投入力度，引导资本投向高新技术产业、现代服务业、能源交通、农村基础设施、环境保护、社会事业等重点领域，着力优化投资结构，提高投资效益，打牢经济社会发展的基础。成立政府金融办公室，积极引进股份制银行和外资银行入驻贵阳，全力支持和推进市商业银行重组上市，组建贵阳农村合作银行。继续加强与亚行、世行、国开行的合作，深化外国政府贷款项目的前期工作，引导社会资本参与经营性的公益事业。组建新的融资平台和融资公司，扩大融资渠道，筹集更多资金投入基础设施建设。鼓励、支持企业运用上市、股权融资、并购重组、发行债券等方式集聚发展要素，加快自身发展。实施积极的创业政策，设立创业投资和风险投资基金，完善扶持创业的金融、信息、市场服务体系，在全社会形成人人乐于创业、人人能够创业的良好氛围。

加大对外开放力度，推动招商引资再上新台阶。一是拓展新的国际交流合作空间。加大与外国使领馆、商业社团的联系，通过外交、商务、文化、旅游、教育交流等渠道努力扩大对外开放，实现更多的交流与合作。二是营造良好的环境。进一步完善招商引资优惠政策，建立健全招商引资责任追究制和激励机制；及时、充分地向社会公布政府的产业布局和政策信息，简化办事程序、提高服务质量，坚决制止吃拿卡要，打造全省一流政务服务中心，为投资者和市民提供高质量、高效率的服务。三是创新招商方式。精心组织好赴西欧、长三角、珠三角、北京、重庆开展大型招商活动，重点在旅游、物流、高新技术产业、铝加工、磷煤化工、基础设施等方面，筛选一批成熟度高、操作性强的项目，采取小分队、专业化、网上推介等多种招商形式，力争引进1—2家世界500强企业、3—4家国内500强企业来筑投资。同时，坚决拒绝能耗高、污染重、占地多、效益低的项目。四是切实抓好项目的跟踪服务工作。继续坚持重大项目领导联系制度和联席会议制度，加强签约项目的督促检查和服务，提高项目的签约率和落地率。

加大扶持力度，引导非公有制经济加快发展。认真落实发展非公有制经济的各项政策，实行“非禁即允”的政策，积极鼓励非公有制企业承包、租赁、收购、兼并国有、集体或其他企业，更多地进入高新技术产业、服务业和社会公益事业等领域。完善中小企业信用担保体系和服务体系，加大对非公有制经济的信贷投入，协调解决好非公有制经济在市场拓展、信息咨询、人才培训和社会保障等方面的实际困难。充分发挥工商联、侨联、台办等机构组织的职能作用，加强对港澳台商、海外投资者、非公有制企业的联系和服务，支持和引导各类商会、行业协会及其它中介组织为非公有制企业搞好服务。规范行政执法，严肃查处损害非公有制企业合法权益的行为。

（六）扎实推进自主创新，加快创新型城市建设步伐

狠抓创新关键环节的突破。强化企业的技术创新主体地位，支持企业建立行业技术联盟，做好高新技术企业和科研机构的引进开发工作，引导和支持创新要素向企业聚集。新建市级企业工程技术中心15家以上，扶持2家市级企业技术中心升级为省级企业技术中心。整合科研院所、高校、军工企业等区域科技力量，启动实施产业关键共性技术、节能减排与可再生能源等重大专项，大力发展民生科技，突破制约全市产业发展的关键技术瓶颈。

积极培育自主品牌和知识产权。继续实施名牌发展战略，加强质量检验检测体系建设，继续抓好省级、市级名牌产品的创牌工作，力争培育2个中国名牌产品。积极做好国家知识产权示范城市创建工作，完善知识产权管理服务体系，加强知识产权保护和专利行政执法，促进专利授权和知识产权保护工作健康发展。引导企业积极采用国际标准和国外先进标准，鼓励企业参与行业、国家和国际标准的制定。

营造良好自主创新环境。完善和落实自主创新促进政策，实行自主创新产品政府采购等激励制度，建立多元化、多层次的自主创新投入体系。加快科技公共服务设施建设，大力发展科技孵化器、技术成果交易平台、创业融资服务机构等，共建“贵阳——以色列创新企业孵化器”，完善贵阳科技大厦孵化平台和科技成果转化功能，启动中国西部（贵阳）高新技术产业研发生产基地建设。在实施贵阳·广州现代中药科技合作项目的基础上，进一步密切两地科技合作交流。省市共建科技风险投资公司，支持科技型企业创业。加强人才队伍建设，实施“高端人才引进计划”，重点引进、培养IT、新材料、先进制造、金融、企业经营管理和生态环保方面的人才，激励各类人才竞相发展。

（七）扎实推进改善民生、服务群众各项工作，着力加快和谐贵阳建设步伐

实施“六有”民生行动计划，夯实生态文明城市建设的民本基础。一是优先发展教育。加大教育投入，对接受农民工子女入学的民办学校同等提供生均公用义务教育经费，多渠道保障进城务工人员子女接受义务教育。巩固提高“两基”水平，完成“两基”迎“国检”各项准备工作，推进教育均衡发展。抓好师德师风建设，加强教师队伍建设特别是农村中小学教师队伍建设，强化示范性高中自主发展能力和辐射带动作用。加快发展高等教育和职业教育。积极支持贵阳学院完善学科体系和培养更多实用人才。加快发展地方性中等职业技术教育，推进贵阳职业技术学院建设，提高职业技术教育为地方经济发展服务的能力。二是加强劳动就业工作。全面落实《就业促进法》，多渠道增加就业岗位，加快就业服务体系向乡（镇）、社区延伸。推进创建“充分就业社区”试点工作，增加公益性就业岗位，加强弱势群体就业援助，保持“零就业家庭”动态为零。完善职业技能培训机制，重点加强被征地农民转移就业培训。实现城乡统筹就业7万人，统筹城乡培训4.4万人。以贯彻《劳动合同法》、规范劳动用工为重点，健全劳动保障监察体制和劳动争议调处仲裁机制，维护劳动者特别是农民工的合法权益。三是发展公共卫生事业。新改扩建息烽妇保院和10个乡（镇）卫生院、127个村卫生室，创建标准示范社区卫生服务中心5个。惠民医疗政策进一步向基层、农村延伸，加快建立大中型医院与社区卫生服务机构双向转诊制度。认真落实重大疾病和高致病性人畜（禽）共患疾病的预防控制措施，严防重大疫情发生和传播扩散。抓好国家“降消项目”和贵阳市第三周期“降低孕产妇死亡率项目”，进一步降低孕产妇和婴儿死亡率及出生缺陷发生率。四是完善社会保障体系。扎实推进养老保险和失业保险市级统筹，确保社保基金安全、规范使用。完善城镇居民和农村人口最低生活保障制度，全面推行城乡医疗救助制度，全面实施城镇职工和居民医疗保险，继续推行新型农村合作医疗制度，确保农业人口参合率稳定在90%以上。稳步提高企业退休人员养老待遇，逐步做实养老保险个人账户。稳步推进新型农村养老保险试点工作。实现养老保险扩面5万人，医疗保险扩面3万人，失业保险扩面2万人，工伤保险扩面3万人，生育保险扩面8万人。进一步完善城乡新型社会救助体系，着力发展以扶老、助残、救孤、济困为重点的社会福利，引导和鼓励社会各界参与慈善活动。五是构建多层次住房供应体系。积极落实调控土地供应和稳定房价的措施，进一步规范房地产市场行为，促进房地产市场健康发展。努力改善中低收入群众住房条件，规范经济适用住房建设和管理，逐步扩大廉租房建设规模，对符合条件的城市低保家庭全面落实廉租住房政策。结合灾后重建工作的开展，积极争取上级资金支持，下大力加快推进农村危房改造，不断提高城乡居民住房保障水平。六是保障社会和谐稳定。大力推进“平安贵阳”工程，深入开展“严打两抢一盗，保卫百姓平安”专项行动，继续抓好“扫黄打非”和禁毒工作，全力维护社会稳定。加强普法宣传、法律援助和司法救助工作，为人民安居乐业提供更加有力的法治保障和法律服务。健全应急救援和指挥系统，增强对突发事件和各类灾害的处置能力。全面落实安全生产责任制，深入开展“隐患治理年”的各项工作，重点加强煤矿、道路交通、建筑施工、社会消防、烟花爆竹、危险化学品、非煤矿山等的隐患排查和专项整治，严防重特大事故发生。切实加强和改进信访工作，进一步完善适应社会转型的利益协调、诉求表达、矛盾调处和权益保障机制，把群众反映的重大信访问题解决在基层，把矛盾化解在萌芽状态。加强食品卫生监督检查，努力保障饮食卫生安全。严肃查处虚抬物价、牟取暴利等不正当行为，适时出台针对困难群体价格上涨的补偿机制，保持物价基本稳定。

大力发展社会各项事业，构建生态文明城市建设的社会平台。一是促进文化大发展大繁荣。实施生态文化建设工程。广泛开展“四城同创”活动，繁荣发展哲学社会科学，深入开展科普工作，提升贵阳文化软实力。加快贵阳数字内容产业园建设，发挥微软技术中心等软件企业的优势，推动软件服务外包和动漫产业发展。办好亚洲青年动漫大赛、第四届药博会、第四届国际阳明文化节、全国汽车拉力锦标赛开阳拉力赛、市第二届旅游发展大会、贵阳温泉节等系列活动，促进文化与会展产业、旅游产业互动发展。推进文化体制改革。完成贵阳日报传媒集团、贵阳京剧院的组建，打造一批有影响的文化品牌。规范文化市场秩序。加大文化市场引导和监督力度，组织开展好全市第三次文物普查，加强对非物质文化遗产和历史街区、文物古迹的保护力度，重点抓好青岩古镇、马头寨、阳明祠、文昌阁等景点的维护修缮工作，彰显具有浓郁地域特色和民族风情的城市人文品质。举办好第五届全市群众文艺调演。加强乡（镇）级文化站和村级文化活动室的建设扶持力度，做好“农家书屋”示范点推广工作，继续实施农村电影放映“2131”工程。推进有线电视网络整合，完成城镇有线电视的数字化整体平移。积极开展“全民健身与奥运同行”和“迎奥运、讲文明、树新风”活动，组织好北京奥运会贵阳境内火炬传递活动，举办好全省首届农民运动会。二是加快社会各项事业协调发展。建立稳定低生育水平长效工作机制，进一步探索完善流动人口计划生育统一管理、优质服务新体制，实行免费婚前医学检查，深层次推进出生缺陷干预工程，提升计生技术服务水平，提高出生人口素质；确保人口自然增长率控制在5.5‰以内。深入开展婚育新风进万家活动和关爱女孩行动，综合治理出生人口性别比偏高。新建4个县级计生技术服务站。全面贯彻执行党的民族宗教政策，按计划推进民族宗教活动场所建设。认真实施妇女儿童发展规划，切实维护妇女儿童的合法权益。加强国防教育，扎实推进“双拥”工作，落实各项优抚政策，巩固军政军民团结。创新外事侨务工作方式，培育、保护和利用侨力资源，进一步扩大对外交流与合作。认真做好统计、保密、档案、文史、修志、老龄、残联等工作。

（八）扎实推进政府建设，着力提升政府执行力和公信力

与时俱进，提高求实创新的能力。切实加强政风建设，牢固树立诚信意识、廉洁意识、服务意识、效率意识。更加注重运用信息技术推动政府管理创新，加强电子政务建设，建立行政审批电子监察系统，增强政府网上办事功能，让全社会普遍享受电子政务的便捷。健全科学民主决策机制。进一步完善重大事项集体决策、专家咨询、社会公示与听证、决策评估制度及决策失误追究制度，提高决策水平。健全政府系统责任管理体系，加快服务型政府建设，强化政府履行社会管理和公共服务的职能。千方百计降低行政成本，确保实现全市行政事业编制、行政公用经费、行政购车经费、行政审批、年审年检项目和行政事业性收费项目“零增长”的目标。

规范行为，提高依法行政的能力。自觉接受人大及其常委会的法律监督和工作监督、政协的民主监督和人民群众以及社会舆论监督。高度重视人大代表、政协委员及各民主党派、工商联、无党派人士的建议、批评、意见，提高建议、提案的办理时效和质量。进一步推进依法行政，规范行政行为。做好行政复议工作，严格实施行政执法责任制和过错追究制。健全政务公开制度、新闻发布制度和重大突发事件快速反应机制，依法保障公民的知情权、参与权、表达权和监督权。

从严治政，提高廉洁自律能力。全面落实党风廉政建设责任制，加大执法监察、效能监察、廉政监察和审计监督的力度，严格执行领导干部任期经济责任审计，健全财政监督检查和财政绩效评估机制，加强对重大工程、产权交易、政府采购等重点领域的管理和监督，严格实行政务公开，强化民主监督。积极开展治理商业贿赂工作，切实从源头上预防和治理腐败，努力建设一支善于学习、忠于职守、清正廉洁、作风优良的公务员队伍。

各位代表，今年年初，我市遭受了有气象纪录以来持续时间最长的一次凝冻灾害。在抗击凝冻的关键时刻，市委、市政府在上级党委、政府的坚强领导下，及时研究和部署“抗凝冻、保民生”工作，各级领导干部深入第一线，全市人民万众一心，社会各界同舟共济，全力以赴做好抗灾救灾的各项工作，确保了人民群众生命财产安全，确保了群众生活正常进行，确保了城市正常运转，确保了社会和谐稳定，取得了抗击凝冻灾害的阶段性重大胜利。当前，我们将按照“目标不变、任务不减”的要求，把“抓重建、保民生”作为政府工作的重中之重，确保全面完成全年目标任务。在抓好灾情核查的基础上，整合各级各类救灾救济资金，重点安排好农村五保户、困难户、受灾户的生产生活，对重灾户提高救灾补助标准，确保每一分救灾救济资金都用在灾区、灾民身上，使受灾群众生产生活得到妥善安置。切实抓好灾区生产恢复工作。加大对农业生产支持力度，认真落实各项支农惠农政策，抓好农资市场检查及平抑物价工作，对因灾导致农作物减收甚至绝收的要“小季损失大季补”，指导农民调整农业结构和抢栽补种，加大养殖小区建设力度、扩大花卉、蔬菜种植面积，做好劳动力培训转移工作，努力将灾害造成的损失补回来，做到农业不减产、不减效，农民不减收。抓好环城林带森林植被修复重建工作，对因灾受损死亡的树木进行全面清理，抢抓季节做好苗木抚育和林木补植补种。这次灾害对电网的破坏直接导致全市工业全面停产，对就业和财政收入造成了较大影响，我们将全力支持、配合电网公司抢修电网，恢复供电，使企业尽快恢复生产。帮助企业协调煤电油运等生产要素，支持企业搞好结构调整和扩能技改，在保证安全的前提下开足马力生产，做到工业产值不降低，工业税收不减少。本着一产、二产损失三产补的原则，大力发展服务业。以“贵阳避暑季”为主体，大力发展避暑经济，吸引更多的游客到我市旅游、观光、消费；加快发展物流、零售和房地产业，使消费在拉动经济增长方面发挥更大的作用。完善防灾减灾体系，加强城乡基础设施建设，使城区及县城、重点乡镇的水、电、气、路、通讯等基础设施逐步达到国家防灾标准。各级政府将在认真总结的基础上，进一步完善应急体系，建立健全应对突发公共事件管理体制机制，进一步加强气象灾害的防御工作，增强对各类灾害、突发公共卫生和公共安全事件等公共危机的预防和处置能力。

各位代表，建设生态文明城市的号角已经吹响，贵阳发展的美好前景已经绘就，我们肩负着历史的重任和全市人民的重托。让我们紧密团结在以胡锦涛同志为总书记的党中央周围，以邓小平理论和“三个代表”重要思想为指导，全面落实科学发展观，深入贯彻落实党的十七大精神，在省委、省政府和市委的正确领导下，在各级人大、政协的监督和支持下，弘扬“知行合一、协力争先”的贵阳精神，奋发图强，加快发展，为把贵阳建设成为生态良好、生产发展、生活富裕、社会文明的生态文明城市而努力奋斗！
